# Soft Foil vs. Adhesive Foil

**Published:** 1872-04

**Authors:** 


### SOFT FOIL *vs.* ADHESIVE FOIL.

One fact appears to be ignored by those who so zealously advocate the use of adhesive foil, which is that equally as good if not better average operations can be performed by first-class, soft foil, hand pressure plugs, and with greatly less fatigue to the patient than with the former material, which is a matter of considerable importance, especially as it is lamentably true that many adhesive foil plugs are now malleted into teeth in such an appalling way as to cause those who have suffered from it to shudder at the thought of having teeth filled.

I do not mean by soft foil plugs those which can be stuffed into teeth at random, and numbering fifteen or twenty a day. No first-class operators, however expert, can accomplish such an amount of good work in ordinary practice, (six or eight being about as much as they will care to undertake)

but I mean cylinders or truncated cones thoroughly consolidated into perfectly prepared cavities, and which finish as beautifully as any adhesive foil plugs and with much less sacrifice of tooth substance. It is well known that those who use adhesive foils are necessarily compelled to cut and file teeth to an alarming extent to secure anchorage for compound operations, because it is impossible to weld adhesive foil successfully except by direct pressure, whereas the skillful soft foil operator accomplishes equally as good results with infinitely less pain and fatigue to the patient, and with less display of material, especially in front teeth. True art ought to conceal art when practicable, and not make people walking advertisements of dentistry.

I can very readily understand why those who are wedded to the use of adhesive foil should be so captivated by the rubber dam it is indispensable as an adjunct in successfully filling with adhesive foil, but with soft foil an unnecessary amount of time would be consumed in its adjustment with no advantage to the operation. The inquiry naturally arises, how would you control the flow of saliva? In most cases, which are not too protracted, hickory wedges at the neck of the tusk in approximate cases, togther with napkins and bibulous paper would be sufficient to keep perfectly dry. However, if, notwithstanding these precautions, the mouth should become flooded (which rarely occurs) it would give me no concern, but I should proceed leisurely without any appliance to complete the operation, for it must be remembered that the integrity of soft foil plugs is not thus impaired, provided the work is thoroughly done. Soft foil operators, it is presumed, do not depend upon the welding or cohesive property of cylinders, but upon lateral pressure in adapting the material to the walls of the cavity, combined with direct pressure in consolidating, which would exclude fluids from the cavity, and if no leakage followed, success would be certain. Time has conclusively proved the truth of this assertion.

Such being the case, why do first class dentists continue to torture patients by making operations "capital when they would be minor if soft foils were employed?" Apparently because they do not understand the art of accomplishing successful results with soft foil, and have not mastered the difficulties attending its constant and daily use in *all approximate cavities.*

Doubtless some will shake their heads and think on reading this, poor fellow, he is ignorant of the advantages of adhesive foils and rubber dams, and mallets, or he would not join issue with prevailing ideas of first-class dentistry. Let such economize their sympathies. I have run the gauntlet of experiment, and now felicitate myself upon the knowledge that results are attainable with soft foil which are impossible of accomplishment with adhesive foil. Teeth can be perfectly plugged without making simple approximate cavities compound, and compound operations can be performed with soft foil made into truncated cones, which are better than cylinders because they have no loose end to perplex the operator. At the same time I recognize at their true value, which is not inconsiderable, the advantages of the aforesaid appliances in exceptional cases, but I still maintain, after a good deal of experience and observation, that first-class soft foil hand pressure plugs will average equally as well, if not better, in preserving teeth than the best adhesive foil plug malleted into teeth to the great discomfort of those who are subjected to such operations.

In conclusion, none of the recent improvements for facilitating the use of adhesive foils, which have come under my personal observation, will in my opinion compensate for ignorance of the virtues of soft foil, and manipulative dexterity in its use. G. P.

